# Serum 25-hydroxyvitamin D and Ethnic Differences in Arterial Stiffness and Endothelial Function

**DOI:** 10.4021/jocmr965w

**Published:** 2012-05-15

**Authors:** Jessica A. Alvarez, Barbara A. Gower, David A. Calhoun, Suzanne E. Judd, Yanbin Dong, Tanja Dudenbostel, Jenni Scholl, Ambika P. Ashraf

**Affiliations:** a Department of Nutrition Sciences, University of Alabama Birmingham, Birmingham, AL, USA; b Vascular Biology and Hypertension Program, University of Alabama Birmingham, Birmingham, AL, USA; c Department of Biostatistics, University of Alabama Birmingham, Birmingham, AL, USA; d Georgia Prevention Institute, Department of Pediatrics, Medical College of Georgia, Augusta, Georgia, USA; e Department of Pediatrics/Division of Pediatric Endocrinology and Metabolism, The Children's Hospital, University of Alabama Birmingham, Birmingham, AL, USA

**Keywords:** Vitamin D, Hypertension, Blood pressure, African Americans, Augmentation index, Pulse wave velocity, Flow mediated dilation, Vascular function

## Abstract

**Background:**

Vitamin D reportedly influences vascular function, which is worse in African Americans (AAs) relative to European Americans (EAs). It is not clear if ethnic differences in 25(OH)D mediate differences in vascular function. This study examined the relationships of serum 25-hydroxyvitamin D (25(OH)D) with indicators of vascular function among healthy, young AA and EA adults.

**Methods:**

This is a cross sectional study involving 23 AAs and 22 EAs. The main outcomes were augmentation index (AIx75), central aortic pressure, pulse wave velocity (PWV), flow-mediated dilation (FMD), and seated and supine blood pressures.

**Results:**

Results indicated that 25(OH)D was inversely associated with AIx75, supine systolic blood pressure (SBP), central aortic SBP and central aortic diastolic blood pressure (DBP), independent of age, sex, and percent body fat (standardized β= -0.29 to -0.43, P < 0.05 for all). AAs had greater AIx75 (P = 0.04) and PWV (P = 0.07) and lower FMD (P = 0.02) compared to EA after adjusting for age and percent body fat; further adjustment for 25(OH)D reduced the ethnic differences (P = 0.44, 0.53, and 0.20, respectively).

**Conclusion:**

The 25(OH)D was associated with vascular function in healthy adults, and lower 25(OH)D among AAs may contribute to their greater arterial stiffness and reduced endothelial function (Clinical trials.gov NCT01041365, NCT01041547).

## Introduction

Increasing evidence suggests a role for vitamin D in the maintenance of cardiovascular health. Several cross-sectional and longitudinal cohort studies have reported relationships between vitamin D deficiencies and the conventional cardiovascular risk factor, hypertension, although vitamin D intervention trials to reduce blood pressure have yielded inconclusive results, as recently reviewed [1]. Fewer studies, however, have investigated the role of vitamin D on non-traditional independent predictors of cardiovascular risk such as arterial stiffness as determined by pulse wave velocity (PWV) and endothelial dysfunction measured by flow mediated dilatation (FMD), particularly in healthy populations [2-7]. Given that endothelial dysfunction and arterial stiffness are considered sub-clinical markers of vascular damage and have been hypothesized to precede the manifestation of hypertension [8, 9], further study investigating the relationships of serum 25-hydroxy vitamin D (25(OH)D) with PWV and FMD in healthy, young adults is warranted.

African Americans (AAs) have a greater prevalence of hypertension and have been shown to have greater arterial stiffness and endothelial dysfunction compared to European Americans (EAs) [10-12]. It is plausible that lower 25(OH)D among AAs relative to EAs [13] may partially account for their relatively poorer vascular health. Indeed, studies have reported a reduction in the ethnic difference in vascular morbidity and mortality after accounting for circulating 25(OH)D [14-17]. Few studies have investigated this hypothesis using intermediate endpoints of vascular disease, such as FMD or PWV [7]. Furthermore, it is not known if relationships between 25(OH)D and these aforesaid vascular outcomes differ by ethnicity.

The purpose of this study was to investigate the relationships of 25(OH)D with indices of vascular function, namely FMD, PWV, and augmentation index (AIx) in healthy AA and EA men and women. We hypothesized that circulating 25(OH)D would be inversely associated with PWV, inversely associated with AIx, and positively associated with FMD. We further hypothesized that lower 25(OH)D among AAs contributes to the ethnic differences in vascular function.

## Methods

### Participants

Participants were 23 AA and 22 EA adults ages 18 - 50 recruited through flyers and newspaper advertisements. Data were collected between February and May 2010. Ethnicity/race was self-defined. Exclusion criteria included self-reported history of diabetes, hypertension, or any other condition known to influence insulin sensitivity or vascular function, such as polycystic ovary syndrome; anti-hypertensive, glucose-controlling, or lipid-lowering medications; monophasic oral contraceptive, hormonal intrauterine device, or injectable contraceptive use; current smoking status; lactose intolerance; or measured BMI > 32 kg/m^2^. Three women were self-classified as postmenopausal. Dietary supplemental use was not reported by any participants. All research was approved by the University of Alabama at Birmingham (UAB) Institutional Review Board for Human Use. Written informed consent (parent consent and subject assent for those less than 19 yrs) was obtained before testing.

### Protocol

Testing was performed over 2 separate days within one week. Fasted blood samples, seated BP, body composition, and anthropometrics were obtained after a 12-hr fast. Radial PWA, carotid-femoral PWV, and FMD testing occurred on a separate morning after an 8-hr fast by a single physician who was blinded to participant vitamin D status.

### Vascular outcomes

#### Seated and supine blood pressure

Seated blood pressure (BP) was measured by a trained research nurse using an electronic auscultatory method (Dinamap Pro 200, GE Medical Systems, Milwaukee, WI). Two measurements were taken within 5 minutes of each other and averaged. A third measurement was taken if systolic BP (SBP) or Diastolic BP (DBP) differed by 5 mmHg or more. Supine BP measurement before vascular function testing was performed after participants rested for at least 5 minutes, using the auscultatory method, with the arm properly supported and using the correct cuff size. BP was measured in both arms, and the average of two readings in the arm with the higher blood pressure reading was used.

#### Pulse wave analysis and pulse wave velocity

Radial PWA and carotid-femoral PWV were performed using the Sphygmocor applanation tonometry system (AtCor Medical, Sydney, Australia) and assessed as previously described [18]. Participants were in the supine position. Briefly, a central aortic pressure waveform was derived from a measured radial artery pressure waveform. AIx was calculated as the difference between the first and second systolic peaks of the ascending aortic waveform divided by the difference between central aortic SBP and central aortic DBP. AIx was standardized to a heart rate of 75 beats/min (AIx75). To determine PWV, ECG-gated pulse pressure waveforms were obtained sequentially over the right carotid artery and the right femoral artery. PWV was calculated as the transit distance between the measurement sites divided by the transit time computed as the time interval between the feet of the two pressure waves.

#### Flow-mediated dilation

FMD was measured non-invasively via high-resolution ultrasound with a 7.5 MHz linear-array probe (Philips HP Agilent Sonos 5500, Andover, MA) after a 30 min rest in the supine position in a quiet, air-conditioned room according to standard guidelines [19]. Briefly, ischemia and reactive hyperemia were induced by inflation of a BP cuff around the forearm to 50 mmHg above the subject’s supine SBP for 5 min followed by rapid deflation. Images of the brachial artery were recorded continuously for 1 min at baseline before cuff inflation and again for 30 sec prior to cuff deflation until 2 min after cuff deflation. Arterial diameter measurements were obtained every 5 cardiac cycles at the end-diastolic cardiac phase for baseline, which was confirmed by the incident R wave on a synchronized electrocardiogram. For peak dilation, every cardiac cycle was measured during reactive hyperemia, and the 5 largest diameters were averaged. FMD was defined as the percentage increase in diameter from baseline to peak dilation.

### Serum analysis, body composition, and anthropometrics

Serum 25(OH)D was measured using liquid chromatography mass spectrometry (Quest Diagnostics, San Juan Capistrano, CA). Vitamin D status was classified as vitamin D insufficient if 25(OH)D concentration was less than or equal to 20 ng/mL. Percentage body fat (% fat) was measured with dual energy X-ray absorptiometry (iDXA, GE-LUNAR Radiation Corp., Madison, WI) with participants lying in the supine position with their arms at their sides. Waist circumference was measured around the narrowest portion of the torso with a flexible tape measure (Gulick II; County Technology, Inc., Gays Mills, WI).

### Questionnaires

SES was measured using the Hollingshead Four-Factor Index of Social Status [20]. A higher score reflects greater SES (scores range from 8 to 66). Physical activity was measured with the validated Beacke Activity Questionnaire; a higher score indicates greater reported physical activity [21].

### Statistical analyses

Descriptive statistics (mean ± SD) were determined for all variables among all participants and by ethnic group. Values greater than 3 SD above or below the mean were removed as outliers. Simple Pearson correlations were used to explore relationships of 25(OH)D with vascular outcomes and age. Vascular outcomes found to be significant or approaching significance were used in subsequent multiple linear regression (MLR) analyses to investigate the relationships between 25(OH)D and vascular outcomes independent of ethnic group, age, and sex. In order to be considered during initial model building, 25(OH)D had to have a P-value of 0.20 or less. Percent body fat and waist circumference were further examined as potential confounders in MLR analyses. Preliminary analyses indicated potential collinearity between ethnic group and 25(OH)D; thus, relationships were also analyzed within each ethnic group. Differences between the ethnic groups were determined using t-tests, chi-squared tests, or analysis of covariance (sequentially adjusting for age, percent body fat, and 25(OH)D concentrations). The distributions of studentized residuals for all MLR models were examined, and residuals greater than 3 SD above or below the mean were considered outliers and subsequently removed from analyses. All tests were two-sided and assumed a 5% significance level. Analyses were performed using SAS software (version 9.1; SAS Institute, Cary, NC).

## Results

Descriptive characteristics of all participants combined and within each ethnic group are shown in Table 1. Participants were generally relatively young (mean age 29.1 ± 6.8 yr) and of normal weight (mean BMI 24.5 ± 4.0 kg/m^2^). A majority of the participants were female (80%). AA tended to be older (P = 0.06), had greater BMI (P = 0.049) and greater percent body fat (P = 0.01) than EA. AA had significantly lower 25(OH)D (P < 0.001), greater AIx (P = 0.005), greater AIx75 (P = 0.002), greater PWV (P = 0.005), and lower FMD (P < 0.05) compared to EA (Table 2). Reported physical activity or SES did not differ between ethnic groups (P = 0.57 and 0.20, respectively). Twenty-three participants (51%) were classified as vitamin D insufficient; of these, 82% were AA.

**Table 1:** Descriptive Characteristics Among all Participants and by Ethnic Group

Variables	All (n = 45)	AA (n = 23)	EA (n = 22)	P_ethnic group_^a^
Age (yr)	29.1 ± 6.8	31.0 ± 6.3	27.1 ± 6.9	0.06
Female gender (%)	80%	87%	73%	0.23
Height (cm)	167.1 ± 7.5	167.4 ± 6.9	166.7 ± 8.3	0.76
Weight (kg)	68.9 ± 13.7	72.6 ± 13.8	65.0 ± 12.8	0.06
BMI (kg/m^2^)	24.5 ± 4.0	25.8 ± 4.2	23.2 ± 3.2	0.02
Waist circumference (cm)	77.0 ± 10.4	78.9 ± 10.8	74.9 ± 9.8	0.20
Percent body fat (%)	30.8 ± 8.0	33.7 ± 8.3	27.7 ± 6.5	0.01
SES total score	41.9 ± 13.5	39.2 ± 12.5^†^	44.5 ± 14.2	0.20
Physical activity score	8.0 ± 1.4	7.9 ± 1.4^b^	8.2 ± 1.4^c^	0.57

Abbreviations: AA, African American; EA, European American; BMI, body mass index. ^a^As measured by two-group t-test. ^b^n = 22, ^c^n = 19, ^†^n = 22

**Table 2:** Serum 25(OH)D and Vascular Measures Among all Participants and by Ethnic Group

Variables^a^	All	AA	EA	P_ethnic group_^b^
25(OH)D (ng/mL)^c^	21.9 ± 10.1	15.2 ± 5.1	28.8 ± 9.4	< 0.001
Vitamin D insufficiency^d^ (n, %)	23 (51%)	18 (82%)	4 (18%)	< 0.001
Seated SBP (mmHg)	110 ± 13	109 ± 12	109 ± 14	0.96
Seated DBP (mmHg)	68 ± 8	70 ± 8	67 ± 7	0.26
Supine SBP (mmHg)	115 ± 11	116 ± 9^e^	113 ± 12	0.36
Supine DBP (mmHg)	72 ± 7	72 ± 6^e^	71 ± 9	0.48
Heart rate (beats/min)	64 ± 8	65 ± 8	62 ± 8	0.18
AIx (%)	10.9 ± 10.2	14.9 ± 9.1	6.6 ± 9.6^f^	0.005
AIx75 (%)	5.6 ± 10.7	10.2 ± 8.1	0.5 ± 11.1^f^	0.002
Aortic SBP (mmHg)	101 ± 9	103 ± 7	99 ± 10	0.08
Aortic DBP (mmHg)	72 ± 8	74 ± 6	71 ± 10	0.26
PWV (m/s)	6.6 ± 0.8	7.0 ± 0.8^e^	6.2 ± 0.7^g^	0.005
Baseline diameter (mm)	0.35 ± 0.05	0.35 ± 0.06^e^	0.34 ± 0.05^f^	0.60
FMD (%)	9.54 ± 2.44	8.61 ± 2.16^e^	10.52 ± 2.39^f^	0.009

Abbreviations: 25(OH)D, 25-hydroxyvitamin D; AA, African American; EA, European American; SBP, systolic blood pressure; DBP, diastolic blood pressure; AIx, augmentation index; AIx75, augmentation index standardized to a heart rate of 75 beats/min; PWV, pulse wave velocity; FMD, flow-mediated dilation. ^a^n = 23 AA, 22 CA unless otherwise specified. ^b^As measured by two-group t-test. ^c^To convert to nmol/L, multiply by 2.946. ^d^Defined as 25(OH)D ≤ 20 ng/mL. ^e^n = 22, ^f^n = 21, ^g^n = 18.

**Figure 1:**
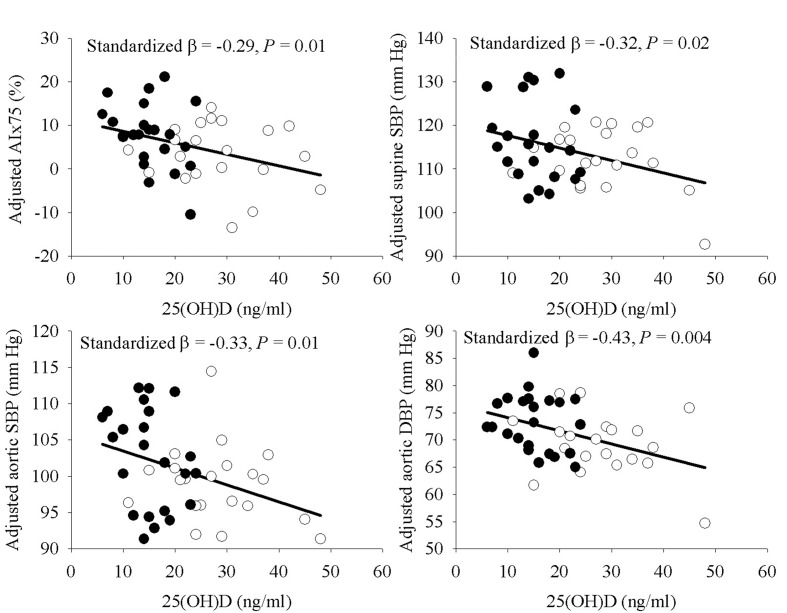
Relationship of 25(OH)D with AIx75, supine SBP, and aortic blood pressures, adjusted for age, sex, and percent body fat. Filled circles indicate AA, open circles indicate EA.

Simple Pearson correlation analyses indicated a significant relationship or a trend towards significance of 25(OH)D with several vascular outcomes, but not seated BP or supine DBP (Table 3). In exploratory MLR analyses for AIx75, supine SBP, and aortic BP as the dependent variables with age, sex, ethnic group, and 25(OH)D as the independent variables, ethnic group was not a significant predictor in the models (P = 0.43 to 0.87) whereas 25(OH)D was either statistically significant or approached significance (P = 0.04 to 0.098). Removal of ethnic group from the models resulted in a significant contribution of 25(OH)D (P < 0.05 for all). As depicted in Figure 1, the relationship of 25(OH)D with AIx75, supine SBP, aortic SBP, and aortic DBP remained statistically significant after incorporating percent body fat to the models. The addition of SES or physical activity to the models did not alter the results (P = 0.009 - 0.04) with the exception of attenuation of supine SBP (P = 0.08) after further adjustment for physical activity. Use of waist circumference or BMI in place of percent body fat as a measure of adiposity yielded similar results, and results were also similar if AIx was used in place of AIx75 (data not shown). The relationship of 25(OH)D with AIx75 was independent of supine and central aortic SBP (data not shown). In MLR models for PWV and FMD, 25(OH)D did not meet the *a priori* criteria for model entry with ethnic group in the models (P = 0.35 and 0.70, respectively), suggesting the relationship between 25(OH)D and these variables was not independent of ethnic group in analyses among the whole group.

**Table 3:** Pearson Correlations of 25(OH)D With Vascular Outcomes (r (P-value))

	25(OH)D
AIx75	- 0.35 (0.02)
PWV	- 0.39 (0.009)
FMD	0.37 (0.01)
Seated SBP	- 0.14 (0.37)
Seated DBP	- 0.11 (0.48)
Supine SBP	- 0.29 (0.06)
Supine DBP	- 0.11 (0.46)
Aortic SBP	- 0.32 (0.03)
Aortic DBP	- 0.27 (0.08)

Abbreviations: 25(OH)D, 25-hydroxyvitamin D; AIx75, augmentation index standardized to a heart rate of 75 beats/min; PWV, pulse wave velocity; FMD, flow-mediated dilation; SBP, systolic blood pressure; DBP, diastolic blood pressure.

### Analyses by ethnic group

The relationships of 25(OH)D with AIX75 (partial r = -0.44, P = 0.05), FMD (partial r = 0.42, P = 0.07), and PWV (partial r = -0.42, P = 0.08) approached significance among AA, but not EA (P = 0.44 to 0.96) (all adjusted for age, sex, and percent body fat). The relationships of 25(OH)D with AIx75 and PWV were independent of supine and central aortic SBP (data not shown). Ethnic differences in the relationships were not seen with the seated, supine, or central aortic BP.

### Contribution of 25(OH)D to ethnic differences in vascular measures

**Figure 2:**
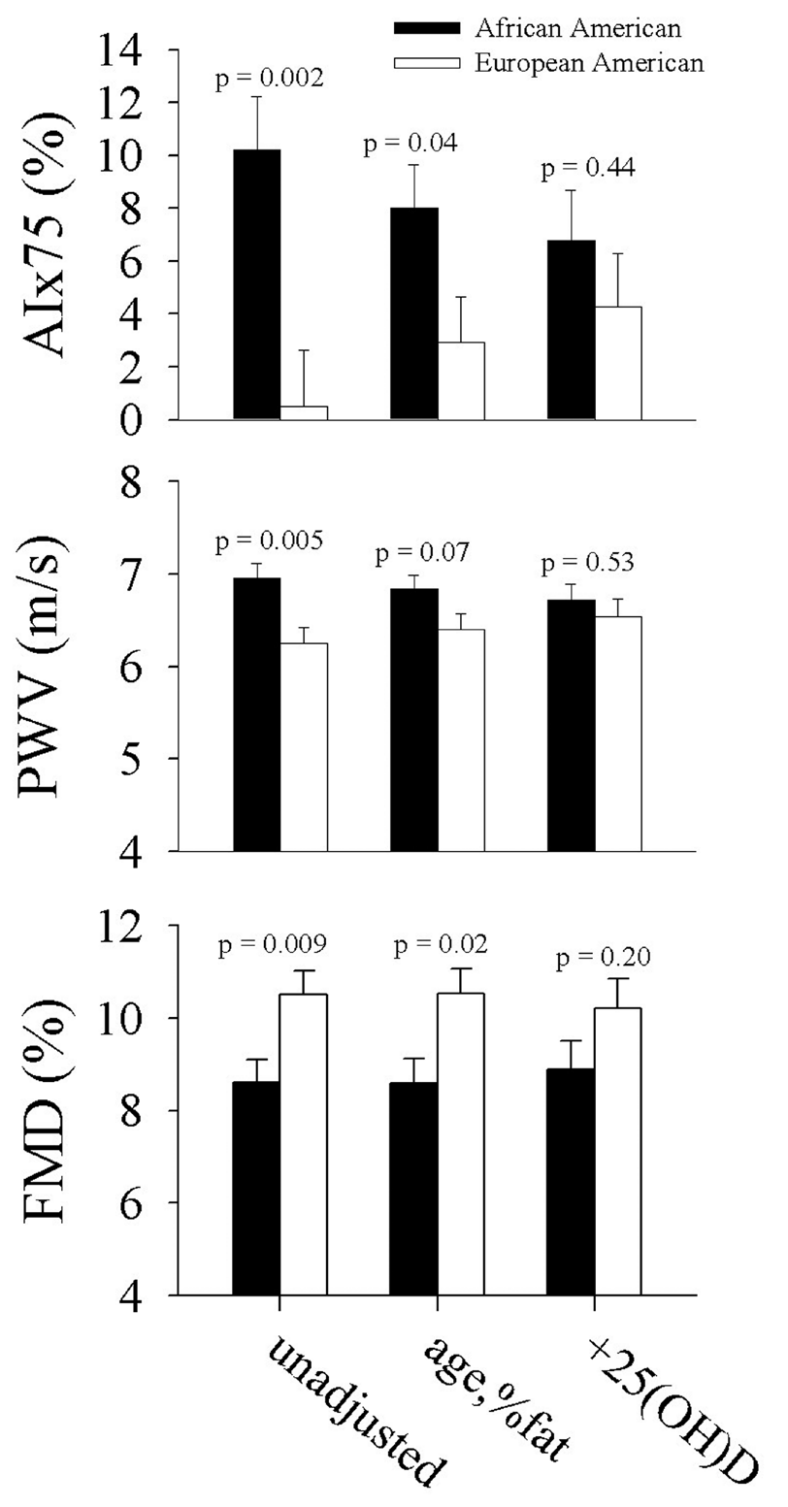
Ethnic differences in vascular function a) unadjusted, b) adjusted for age and percent body fat, and c) further adjusted for 25(OH)D.

Ethnic differences in AIx75, PWV, and FMD were attenuated after adjustment for age and percent body fat, and they were further attenuated after additional adjustment for 25(OH)D (Fig. 2). There was a 93% difference between the ethnic groups in AIx75 after adjusting for age and percent fat (P = 0.04); further adjustment for 25(OH)D reduced the ethnic difference to 45.1% (P = 0.44). For FMD, there was a 20.3% difference between the ethnic groups after adjusting for age and percent fat (P = 0.07) which was reduced to a 13.8 % difference after additional adjustment for 25(OH)D (P = 0.53). The ethnic difference in PWV was 6.8% (P = 0.02) after adjustment for age and percent fat, and it was reduced to a 2.9% ethnic difference after further adjustment for 25(OH)D (P = 0.20).

## Discussion

In this study, we investigated the relationship between circulating 25(OH)D and a comprehensive assessment of vascular function in apparently healthy AA and EA adults. Results indicated that 1) 25(OH)D was associated with several non-traditional indices of vascular function but not with traditionally-measured BP; however, the strength of the relationships with some indices were stronger in AA than in EA; and 2) there were ethnic differences in several vascular function indices; however, adjustment for 25(OH)D reduced these ethnic differences. Taken together, these results indicate a relevant role for vitamin D in vascular health, particularly among AA, a group with a disproportionate risk for CVD.

In our study, 25(OH)D was inversely and independently associated with central aortic BP, but it was not significantly associated with the traditionally-measured brachial BP. Many studies have examined the relationship between 25(OH)D and BP using the latter measurement; the results have been conflicting [15, 22, 23]. To our knowledge, we are the first to investigate the relationship between 25(OH)D and central aortic blood pressure. Our findings suggest brachial BP, at least in a young adult population, underestimates the true relationship between 25(OH)D and BP. NHANES III analyses have suggested that linear relationships of 25(OH)D with SBP are stronger in elderly (ages 65 yr and older) compared to younger populations (< 50 yr)[23]. Thus, it is possible that with increasing age, relationships between 25(OH)D and brachial BP become more apparent.

Circulating 25(OH)D was inversely associated with AIx, independent of ethnicity and body composition, in our participants. AIx is an assessment of arterial wave reflection, which is influenced by both peripheral small artery structure/tone and PWV [24]. Our data thus support a role of vitamin D in arterial pressure wave reflection and concur with findings in patients with kidney disease [25], as well as recent findings in a separate cohort of healthy adults [6]. Future studies should investigate whether vitamin D intervention reduces AIx.

We found, in agreement with others [11, 12], that AIx and PWV were higher and FMD was significantly lower in AA compared to EA, independent of differences in body composition. Additionally, 25(OH)D is lower in AA [13]. Rezai et al [7] report that 25(OH)D accounts for ethnic differences in PWV among South Asians compared to African Caribbeans and Europeans. We show that among AA and EA, adjustment for differences in 25(OH)D reduces the ethnic differences in AIx, PWV, and FMD, suggesting lower 25(OH)D among AA at least partially accounts for their poorer vascular function compared to EA. This was particularly evident for AIx. Similarly, others have suggested that lower 25(OH)D in AA mediated ethnic differences in BP [14, 15]. Collectively, these data support previous findings that 25(OH)D may contribute to greater prevalence of peripheral vascular disease and cardiovascular morbidity and mortality in AA [16, 17].

We analyzed the relationships of 25(OH)D with vascular measures within each ethnic group because of the potential confounding of low 25(OH)D and poorer vascular function reduced arterial compliance among AA compared to EA. Results suggested that 25(OH)D was associated with AIx, PWV, and FMD in AA, but not in EA. The sample sizes precluded sufficient power to obtain statistically significant results; thus these findings can only be considered hypothesis-generating. It is possible that the disparate relationships between 25(OH)D and vascular function may be a result of differing phenotypes at the cellular/molecular level of endothelial cells [26]. As AA have superior bone quality and calcium economy compared to EA [27], it is also conceivable that vitamin D acts preferentially at bone in EA and at non-skeletal tissues in AA. Vitamin D supplementation significantly reduced PWV in AA adolescents [5] and increased FMD [4] in AA adults, however vitamin D supplementation trials aimed at PWV or FMD have not been performed in healthy, EA populations. Thus, future randomized and controlled trials including sufficient sample sizes of both EA and AA are warranted. The current study also underscores the difficulties of assessing cross-sectional relationships of 25(OH)D with health outcomes in mixed ethnic group cohorts where there are differences in both 25(OH)D and health outcomes.

There are many potential mechanisms mediating a vitamin D effect on vascular function. Vascular smooth muscle and endothelial cells express vitamin D receptors and 1α-hydoxylase [3, 28], allowing for autocrine production of 1, 25-dihydroxyvitamin D (the biologically active form of vitamin D), which has been shown to regulate various cellular processes, including cell proliferation [29]. Other potential mechanisms linking vitamin D to vascular health are decreases in oxidative stress [2], attenuation of NF-κB activation and subsequent inflammation [3], reduction of parathyroid hormone [30], and/or down regulation of the renin-angiotensin-aldosterone system [22].

A strength of the study is the investigation of 25(OH)D relationships with a comprehensive assessment of vascular function. Furthermore we used a robust measure of body composition to account for potential confounding effects of adiposity. The cross-sectional design precludes cause-and-effect inferences. It is yet to be established if alteration of these relatively novel measures of vascular function reduces CVD risk. We did not assess mechanistic pathways through which 25(OH)D could be cardio-protective, such as the renin-angiotensin system or parathyroid hormone. The small sample size limited the number of potential confounders that could be included in analyses and our ability to obtain statistically significant results in some sub-group analyses. Finally, it is possible that these findings may not be generalizable to other ethnic or age groups or groups of different health status.

### Conclusion

Circulating 25(OH)D was associated with various relatively novel indicators of vascular function, including arterial stiffness and endothelial function. Efforts to improve vitamin D status may be particularly relevant in AA, as the relationships appeared to be stronger in this ethnic group compared to EA. Long-term, randomized, placebo-controlled trials investigating vitamin D supplementation effects on vascular function among multiple ethnic groups are warranted.

## Clinical Trials

NCT01041365 (12/28/2009); NCT01041547 (12/28/2009).

## Abbreviations

25(OH)D: 25-hydroxyvitamin D
AAs: African Americans
AIx: augmentation index
AIx75: augmentation index adjusted for a heart rate of 75 beats/min
BP: blood pressure
DBP: diastolic blood pressure
EAs: European Americans
FMD: flow-mediated dilation
MLR: multiple linear regression
NF-κB: nuclear factor-KappaB
PWA: pulse wave analysis
PWV: pulse wave velocity
SES: socioeconomic status
SBP: systolic blood pressure
UAB: University of Alabama at Birmingham
